# Supplementary material for: Novel Driver Strength Index highlights important cancer genes in TCGA PanCanAtlas patients
Source: PeerJ. 2022 Aug 11;10:e13860. doi: 10.7717/peerj.13860 (PMC9375969; doi:10.7717/peerj.13860)
Supplement: Supplemental Information 2 [file peerj-10-13860-s002.zip › COHORTS/genes plots/detailed/2021_8_16_14_9_distribution_events_detailed_females_29.pdf]

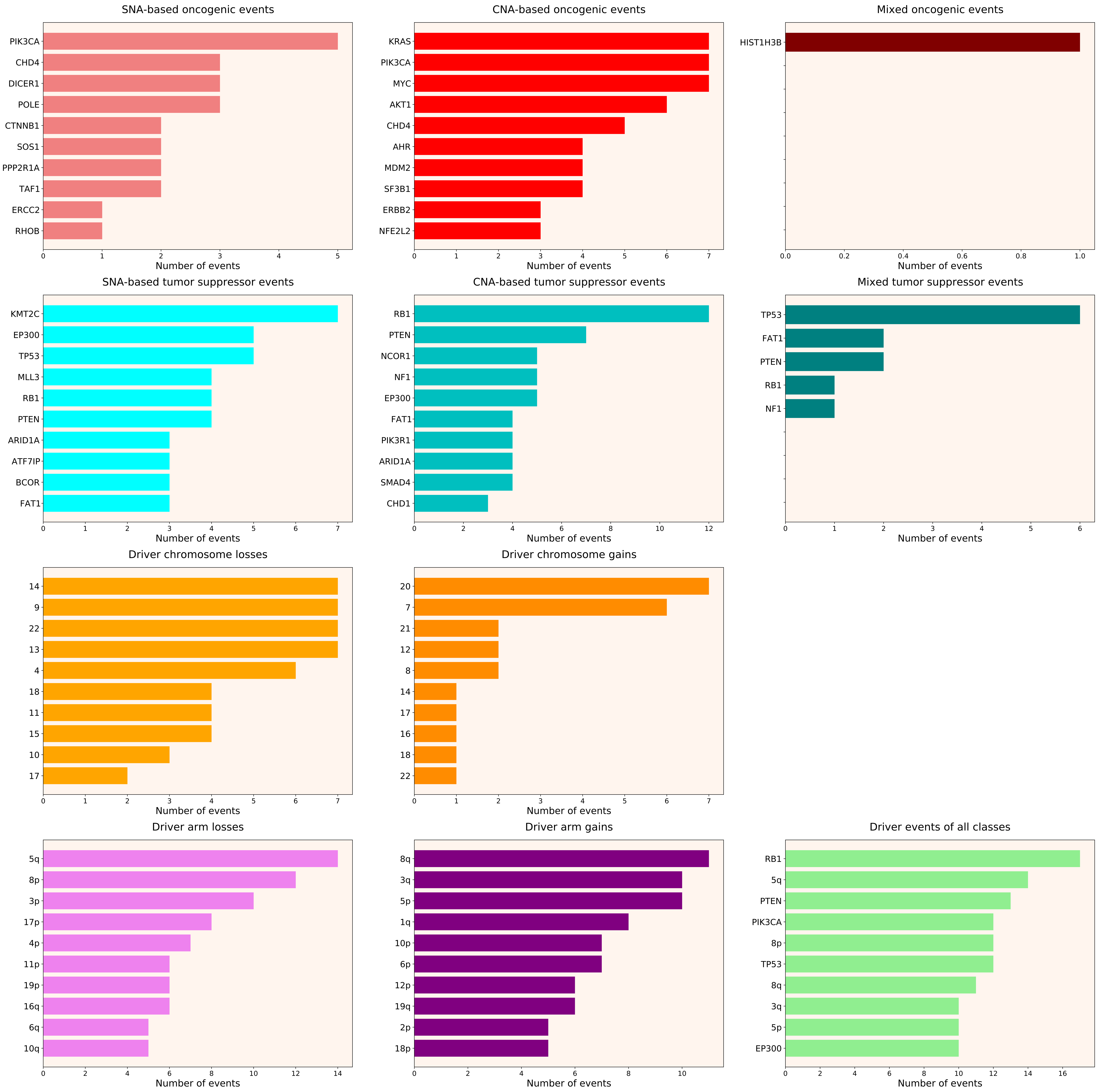
SNA-based oncogenic events
PIK3CA
CHD4
DICER1
POLE
CTNNB1
SOS1
PPP2R1A
TAF1
ERCC2
RHOB
Number of events
CNA-based oncogenic events
KRAS
PIK3CA
MYC
AKT1
CHD4
AHR
MDM2
SF3B1
ERBB2
NFE2L2
Number of events
Mixed oncogenic events
HIST1H3B
Number of events
SNA-based tumor suppressor events
KMT2C
EP300
TP53
MLL3
RB1
PTEN
ARID1A
ATF7IP
BCOR
FAT1
Number of events
CNA-based tumor suppressor events
RB1
PTEN
NCOR1
NF1
EP300
FAT1
PIK3R1
ARID1A
SMAD4
CHD1
Number of events
Mixed tumor suppressor events
TP53
FAT1
PTEN
RB1
NF1
Number of events
Driver chromosome losses
14
9
22
13
4
18
11
15
10
17
Number of events
Driver chromosome gains
20
7
21
12
8
14
17
16
18
22
Number of events
Driver arm losses
5q
8p
3p
17p
4p
11p
19p
16q
6q
10q
Number of events
Driver arm gains
8q
3q
5p
1q
10p
6p
12p
19q
2p
18p
Number of events
Driver events of all classes
RB1
5q
PTEN
PIK3CA
8p
TP53
8q
3q
5p
EP300
Number of events
